# The role of colonic motility in low anterior resection syndrome

**DOI:** 10.3389/fonc.2022.975386

**Published:** 2022-09-16

**Authors:** Chris Varghese, Cameron I. Wells, Ian P. Bissett, Gregory O’Grady, Celia Keane

**Affiliations:** ^1^ Department of Surgery, Faculty of Medical and Health Sciences, University of Auckland, Auckland, New Zealand; ^2^ Department of General Surgery, Counties Manukau District Health Board, Auckland, New Zealand; ^3^ Department of Surgery, Auckland City Hospital, Auckland, New Zealand; ^4^ Department of Surgery, Whangārei Hospital, Whangarei, New Zealand

**Keywords:** low anterior resection syndrome (LARS), colonic motility, rectosigmoid brake, rectal cancer, low anterior resection

## Abstract

Low anterior resection syndrome (LARS) describes the symptoms and experiences of bowel dysfunction experienced by patients after rectal cancer surgery. LARS is a complex and multifactorial syndrome exacerbated by factors such as low anastomotic height, defunctioning of the colon and neorectum, and radiotherapy. There has recently been growing awareness and understanding regarding the role of colonic motility as a contributing mechanism for LARS. It is well established that rectosigmoid motility serves an important role in coordinating rectal filling and maintaining continence. Resection of the rectosigmoid may therefore contribute to LARS through altered distal colonic and neorectal motility. This review evaluates the role of colonic motility within the broader pathophysiology of LARS and outlines future directions of research needed to enable targeted therapy for specific LARS phenotypes.

## Introduction

Rectal cancer prevalence is increasing, particularly in those under 50 (1), with surgery and radiotherapy as the mainstays of curative treatment. There have been substantial improvements in oncological outcomes and permanent stoma rates attributable to total mesorectal excision, neoadjuvant radiotherapy, circular stapling devices, multidisciplinary team management, and multimodal treatment approaches (2–4). These improvements have unmasked a high prevalence of ‘survivorship’ disease, particularly disordered bowel function that significantly impairs patients’ quality of life (5). As colorectal cancer survivorship continues to increase worldwide with improved screening programmes, earlier detection, and novel treatment advances (6), these ‘survivorship’ consequences have become important clinical and research priorities (1, 7, 8).

Several terms have been used to describe these survivorship disorders such as ‘pelvic radiotherapy induced dysfunction’, and ‘low anterior resection syndrome’. Recently, the symptoms and consequences of bowel dysfunction that patients suffer after rectal resection, termed low anterior resection syndrome (LARS), have been defined and standardised based on international consensus (9). LARS is a highly prevalent outcome affecting up to 44% of patients after low anterior resection (10, 11), and can be long-standing, often persisting up to 18-months after surgery (12). LARS is consistently associated with poor quality of life, and as access to rectal cancer treatment improves, represents a growing burden of disease (5, 10, 11, 13). Despite this significant patient- and healthcare burden associated with LARS, much remains to be clarified regarding the pathophysiology of LARS.

In the setting of increasingly minimally invasive surgery, chemoradiotherapy, and emerging watch and wait approaches (14, 15), surgical attention is beginning to focus on methods to mitigate LARS. This is complicated by the fact that the pathophysiology of LARS is multifactorial and complex (16). The MANUEL project (16) provides the best current guidance on the management of LARS, advocating for therapies directed based on proposed disease mechanisms (16). Clarifying these disease mechanisms and presenting objective biomarkers will be needed to improve the prevention and therapy of LARS.

The heterogenous symptoms experienced by patients with LARS likely also reflects the multifactorial pathophysiology of the syndrome. Surgical trauma to the anal sphincter complex, colonic denervation, reduced neorectal capacity and compliance, radiotherapy-induced fibrosis, and faecal diversion, have all been shown to negatively impact bowel function after rectal resection (12, 17–20). While anorectal factors have been extensively researched with regards to their role in LARS (17, 21–24), less is known about the role of colonic physiology in the aetiology of LARS. Research has continued to emerge that significant alterations in colonic motility are present in LARS, likely demonstrating an underappreciated pathophysiological mechanism (25, 26). This review will focus on this important role of colonic motility and its implications in LARS.

## An overview of LARS

### Diagnosis and symptoms of LARS

The term LARS has historically been used to report heterogeneous symptomatology often with a focus on faecal incontinence, neglecting other symptoms shown to be more strongly associated with patients’ postoperative quality of life (27). A recent international patient-provider initiative established a consensus-based definition for LARS (9). Variable or unpredictable bowel function, altered stool consistency, increased stool frequency, repeated painful stools, emptying difficulties, urgency, incontinence, and soiling were identified as the most important symptoms of LARS, according to real-world patient experience. A diagnosis of LARS can be made when patients experience at least one of these symptoms which results in one of the following consequences: toilet dependence, preoccupation with bowel function, dissatisfaction with bowels, strategies and compromises, impact on; mental and emotional wellbeing, social and daily activities, relationships and intimacy, and/or roles, commitments and responsibilities (9). This standardised definition enables consistent reporting of LARS and therefore robust investigation into LARS pathophysiology.

### Measurement of LARS

Accurately measuring LARS in a standardised manner is an essential step to better understanding the pathophysiology of LARS. Historically, a wide range of validated and unvalidated tools have been used to report LARS, further contributing to the complexity of assessing this syndrome (27). The best currently available tool is the LARS score (28). While the LARS score has been widely adopted due to its simplicity, it suffers from several limitations; including inability to adequately capture evacuatory dysfunction, over- and under-estimation of impact on some patients’ quality of life due to lack of a self-reported quality of life metric, and inadequacy to monitor treatment efficacy due to insensitivity to change (16, 29). A new severity scoring tool, based upon the consensus definition of LARS is currently under development, which aims to more accurately capture patient experience and may therefore enable a more nuanced investigation of the various pathophysiological mechanisms for LARS.

### Treatment of LARS

Current management of LARS is reactive, empirical, and symptom based (30). Management guidelines are based on expert opinion due to a sparse evidence base, with evidence of therapeutic efficacy often extrapolated from literature based on studies done in cohorts with a native rectum (16, 31). A hierarchical approach is advocated, with the majority of patients requiring non-invasive intervention such as dietary manipulation, pharmacological intervention, or pelvic floor rehabilitation (16, 30, 31). There has traditionally also been a focus on faecal incontinence or urgency without a wider appreciation of the broad range of defecatory dysfunctions that can be seen in LARS (31). This in part stems from the difficulty elucidating the complex pathophysiological mechanisms underlying LARS and in part from many clinicians’ misguided perception that incontinence has the greatest impact on patients’ quality of life (32). Further improvements in management will rely on appropriate patient selection, suitable outcome measures to assess treatment efficacy, and treatments rationalised to mechanisms of disease.

## Colorectal anatomy and physiology

### Colon anatomy and physiology

The motility of the colon and rectum are mainly controlled by overlapping neurogenic and myogenic systems, modulated by local mucosal factors and hormonal influences (33, 34). Like the stomach and small bowel, the colon contains networks of interstitial cells of Cajal (ICC), which aid in the generation of rhythmic motor patterns under co-regulation by intrinsic and extrinsic (autonomic) nervous systems (34, 35). ICC found in the submuscular plexus (SMP) are the primary pacemakers in the colon, these intercalate with intermuscular ICC with closer proximity to nerve varicosities supporting the importance of extrinsic neural control (36, 37). The anatomy and functioning of ICC in the colon have been comprehensively outlined in a recent review (34). Parasympathetic supply to the distal colon is by S2-S4 pelvic splanchnic nerves, and the sympathetic supply *via* the L1-L2 splanchnic nerves (emanating from the inferior hypogastric plexus). The sympathetic nerves inhibit proximal colonic motility tonically (38), but are excitatory to the internal anal sphincter (IAS).

### Rectal anatomy and physiology

**Figure 1:**
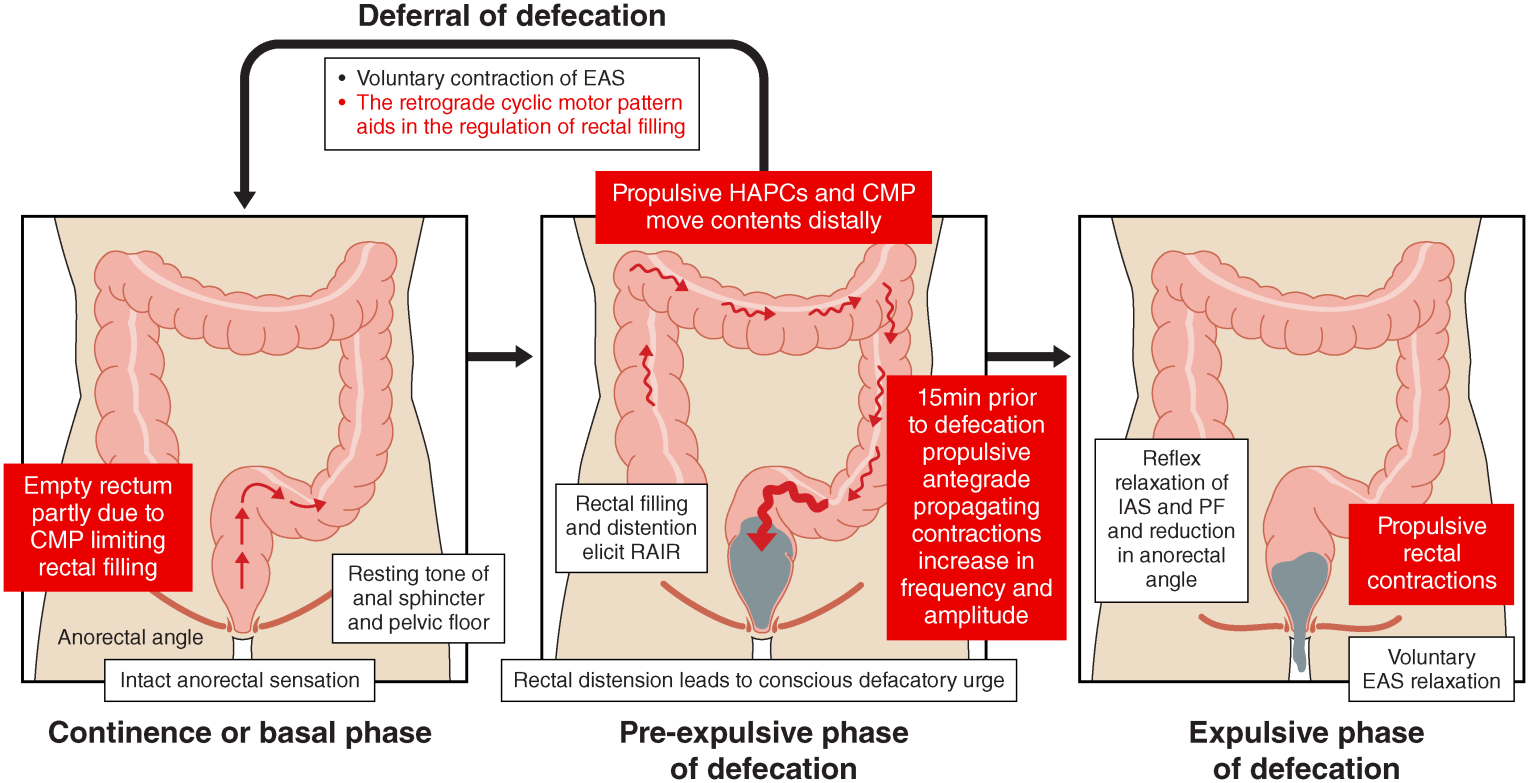
Physiological mechanisms of defecation with a focus on the role of colonic motility. The cyclic motor pattern (CMP) which characterises the rectosigmoid brake regulates rectal filling in the continence/basal phase. During the pre-expulsive phase of defecation, high amplitude propagating contractions (HAPCs) and the cyclic motor pattern facilitate antegrade transit. Important mechanisms of continence include ability of voluntary contraction of the external anal sphincter (EAS) and the rectosigmoid brake. The expulsive phase of defecation is facilitated by propulsive rectal contractions, voluntary EAS relaxation, and reflex relaxation of the internal anal sphincter (IAS) and pelvic floor (PF) muscles. Refer to refs (16, 44) for extended details.

The rectum lies between the sacral promontory and anus, measuring between 12-15 cm, and has an important role in continence. The empty rectum is filled with faeces by antegrade colonic propagating contractions (39). High amplitude propagating contractions (HAPC) which start at the ascending colon and propagate as far as the sigmoid colon, have been associated with stool entering the rectum, causing an increase in intrarectal pressure, activation of the recto-anal inhibitory reflex, and relaxation of the IAS. External anal sphincter (EAS) and pelvic floor muscle tone then contribute to the maintenance of continence. The role of the cyclic motor pattern has been reviewed previously (40), but in brief: the cyclic motor pattern originates throughout the distal colon, most frequently at the rectosigmoid junction, and predominantly propagating in the retrograde direction after meals, thereby preventing rectal filling and aiding in bowel continence (40–42). Voluntary passage of flatus and anorectal differentiation of gaseous, liquid, and solid contents; important functions for the voluntary control of defecation, are mediated through the recto-anal inhibitory reflex whereby rectal distension causes transient internal sphincter relaxation before recovery of anal pressures (43). A conscious defecatory urge arises with progressive rectal distension and defecation is achieved through voluntary relaxation of the EAS, involuntary contraction of the IAS, reduction in the anorectal angle and propulsive rectal contractions (see **Figure 1**) (44).

### Colonic motility in health

Distal colonic motility has long been considered important for bowel function and continence, with particular attention given to the rectosigmoid region. As early as the 1920s, it was posited that sigmoid motility patterns play an important role in the control of defecation by limiting rectal filling (45, 46), a mechanism that is normally suppressed during defecation. In a kymographic study of 18 symptomatic individuals, a reduction in sigmoid motility was associated with diarrhoea (47). Building on this, O’Beirne described the rectosigmoid region as a ‘functional sphincter’ (48), and Rao and Welcher further proposed that periodic rectal motor activity served as an ‘intrinsic braking mechanism to prevent the untimely flow of contents’ (42).

Several groups have comprehensively characterised HAPCs, which as noted above, are often associated with defecation (49–52). While HAPCs are the most widely recognised colonic motility pattern, they are not the most dominant colonic motor pattern. Using high-resolution colonic manometry (HRCM), which allows for comprehensive spatiotemporal assessment of motility patterns, Dinning et al. showed that the cyclic motor pattern (CMP) is the most active colonic motility pattern over time (53). The CMP is a repetitive sequence of propagating contractions with a frequency of 2-6 cycles per minute (cpm). In a cohort of healthy controls, the CMP lasted a mean of 12.6 ± 2.9 s, had a mean amplitude of 23.1 ± 21.4 mmHg, increased post-prandially, and propagated predominantly in a retrograde direction (53). Lin et al. extended this work and identified that the majority of CMP originate from the rectosigmoid region (54). An increase in the retrograde CMP in response to a meal-stimulus was considered a physiological marker of a rectosigmoid brake, limiting rectal filling and aiding in the maintenance of bowel continence (40). Chen et al. also characterised rectosigmoid motility patterns using similar HRCM methods, further describing an intermittent pressure band 10-17 cm above the anal verge that was described as relaxing and contracting in concert with the anal sphincters (54, 55). These data appear to confirm the earlier hypotheses of a “functional sphincter” in this region (48), with this constellation of distal motility profiles collectively considered to represent the rectosigmoid brake. In addition to the comprehensive HRCM profiling of rectosigmoid motility patterns, multiple modalities have verified the function of this region of the colon. For example, high-resolution impedance manometry demonstrated that gas insufflation of the sigmoid colon can initiate retrograde CMP which in turn limits gas transit into the rectum, further supporting the rectosigmoid brake hypothesis (56).

### Mechanism and regulation of the rectosigmoid brake

As discussed above, much of the periodic gastrointestinal motility relies on spontaneous oscillatory depolarisation of smooth muscles typically patterned by ICC. It is thought that the ‘slow-waves’ predominantly generated by ICC in the colon arise in the submucosal layer, in concert with extrinsic neuronal input as a cooperating mechanism, together governing the cyclic motor pattern (57, 58). An extensive overview of the role of ICC in regulating colonic function has previously been provided by Huizinga et al. (34) Neural input, thought to co-regulate the CMP, is primarily from the enteric nervous system but is also modulated by the parasympathetic and sympathetic nervous systems.

The importance of neural innervation in regulating rectosigmoid motor activity is evidenced by its relative absence in spinal cord injury (59, 60), systemic sclerosis (61), and diabetes mellitus (62). Similarly, as stimulation of the pelvic splanchnic nerves through sacral neuromodulation (SNM) can be highly efficacious even in cases of anal sphincter incompetence (63, 64), it has also been posited that neurally-mediated colonic motility pathways are a primary mechanism for regulating continence *via* SNM (41, 65).

### Pathophysiology of a dysregulated rectosigmoid brake

Rectosigmoid dysmotility may be associated with disrupted continence. In a recent HRCM study, a suppressed rectosigmoid brake (i.e., fewer retrograde propagating contractions in the distal colon) was observed in patients with medically-refractory faecal incontinence compared to healthy controls (41). While this study was in participants with a native rectum, it is conceivable that a similar attenuation of the rectosigmoid brake may contribute to incontinence and soiling in LARS (41). Numerous studies have demonstrated that sacral neuromodulation (SNM), an established and highly successful treatment for faecal incontinence, upregulates retrograde motility of the sigmoid colon, restoring rectosigmoid brake function (41, 65, 66). Successful application of SNM to relieve symptoms in patients with LARS could also function through this mechanism, by upregulating propagating sequences with a braking function in the remnant colon or neorectum (67).

Interestingly, a hyperactive rectosigmoid brake has also been recently hypothesised to contribute to constipation, by impeding the normal passage of bowel motions through the distal colorectum (68–70). Our group observed this effect in a patient who underwent first stage SNM for incontinence but failed to progress to a permanent implant due to the new onset of constipation. Interestingly this patient demonstrated increased baseline propagating activity with SNM (41). A similar phenomenon is seen after surgery where a hyperactive rectosigmoid brake is thought to impair recovery of bowel function, and resection of the rectosigmoid has therefore been proposed as a plausible explanation for the faster bowel recovery after distal colectomy *vs* right-sided resections (71–73). Parasympathetic denervation of the distal colon and neorectum may cause similar hyperactivity in patients with LARS, particularly in those patients with evacuatory dysfunction predominant symptoms.

## Colonic motility after anterior resection

**Figure 2:**
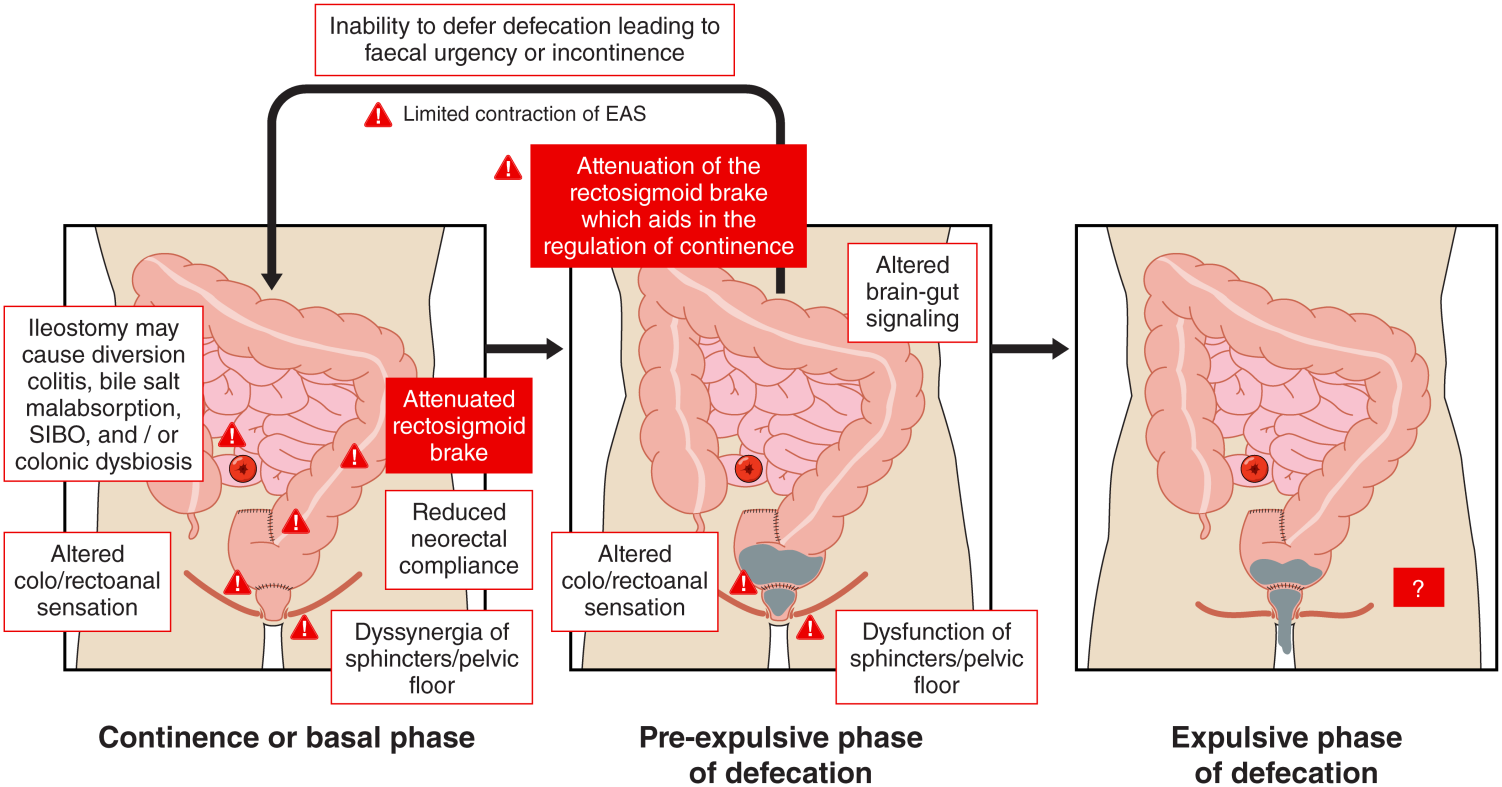
Putative mechanisms contributing to low anterior resection syndrome during the basal, pre-expulsive, and expulsive phases. Mediators of injury arising from surgery, radiotherapy, and ileostomies (as described in **Figure 5**) are described in this figure. Diverting ileostomies may cause diversion colitis, bile salt malabsorption, small intestinal bacterial overgrowth (SIBO), and/or colonic dysbiosis. Similarly, changes in colo/rectoanal sensation, neorectal compliance, sphincter, and pelvic floor dyssynergia, altered distal colonic motility, and limited external anal sphincter (EAS) function also contribute. Little is known about the pathophysiological mechanisms for low anterior resection syndrome during the expulsive phase of defecation and more research is required in this area.

Resection of the rectosigmoid during anterior resection is hypothesised to result in aberrant distal colon and neorectal motility *via* resection of the rectosigmoid brake and denervation of the remnant colon and neorectum (**Figure 2**). High resolution colonic (HRCM) manometry and barostat studies have demonstrated an altered meal response or gastrocolic reflex in patients after anterior resection compared to healthy controls, consistent with anecdotal patient reports of a temporal association between eating and symptom onset.

Our research group has investigated post-anterior resection colonic motility using HRCM in multiple cohorts. Vather et al. report a comparison between 15 patients post-resection, who reported “normal” bowel function according to the comprehensive faecal incontinence questionnaire (CFIQ) and 9 healthy controls (74). Keane et al. report comparisons between 11 patients post-resection who report no LARS according to the LARS score, 12 patients with LARS, and the same 9 healthy controls (25). The analysis methods differ with visual inspection in the former study and automated analysis with a high sensitivity (5 mmHg amplitude threshold) in the latter. Both studies found a largely conserved meal response post-resection in patients with “normal bowel function” but a reduced post-prandial distance of propagation for antegrade and retrograde propagating sequences (25, 74). Interestingly though, the more sensitive analysis also revealed fewer post-prandial retrograde contractions compared with controls (25). Recently, Ansong et al. performed HRCM in 9 patients with no/minor LARS and 9 patients with major LARS and found major LARS was associated with increased antegrade CMP which correlated with symptoms, and fewer bisacodyl-stimulated HAPCs. The increased antegrade CMP persisted after bisacodyl stimulus (75). The authors postulate this may relate to other findings of increased colonic transit in LARS. The vast heterogeneity seen in these groups with preserved bowel function post-resection highlights that the mechanisms underlying post-operative bowel function recovery and dysfunction are complicated. Excision of the native rectosigmoid is likely associated with numerous compensatory mechanisms to mitigate some of the effects of an attenuated rectosigmoid brake.

**Figure 3:**
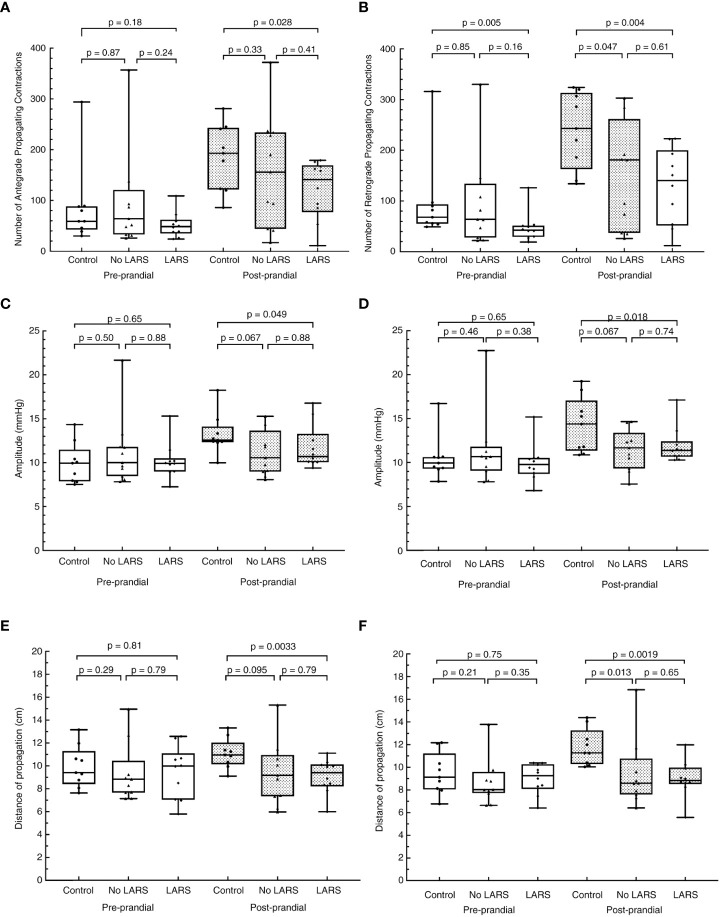
Adapted from Keane et al. with permission. Differences in the cyclic motor pattern in low anterior resection syndrome (LARS) compared to post-low anterior resection patients without LARS, and healthy controls. **(A)** number of antegrade contractions, **(B)** number of retrograde contractions, **(C)** amplitude of antegrade propagating contractions, **(D)** amplitude of retrograde propagating contractions, **(E)** distance of propagation of antegrade contractions, and **(F)** distance of propagation of retrograde propagating contractions. The resection group is made up of patients who scored ≤20 on the LARS score.

HRCM demonstrated attenuation of the rectosigmoid brake mechanism in the patients with LARS, as they had fewer retrograde propagating contractions pre- and post-meal, which propagated for a shorter distance with reduced amplitude post-meal, compared with healthy controls (**Figure 3**) (25). However, attenuation of the retrograde rectosigmoid motility doesn’t appear to be the sole mechanism responsible, as patients with LARS also had fewer post-prandial antegrade contractions, which propagated for a shorter distance with reduced amplitude compared to healthy controls (**Figure 3**) (25). Therefore removal of this region may have additional effects on long feedback loops within the gastrointestinal system as well as impacts on neorectal filling. The main limitations of the HRCM work are the small samples and the lack of contemporary comprehensive anorectal physiology testing to distil the various mechanisms contributing to dysfunction in individual patients.

**Figure 4:**
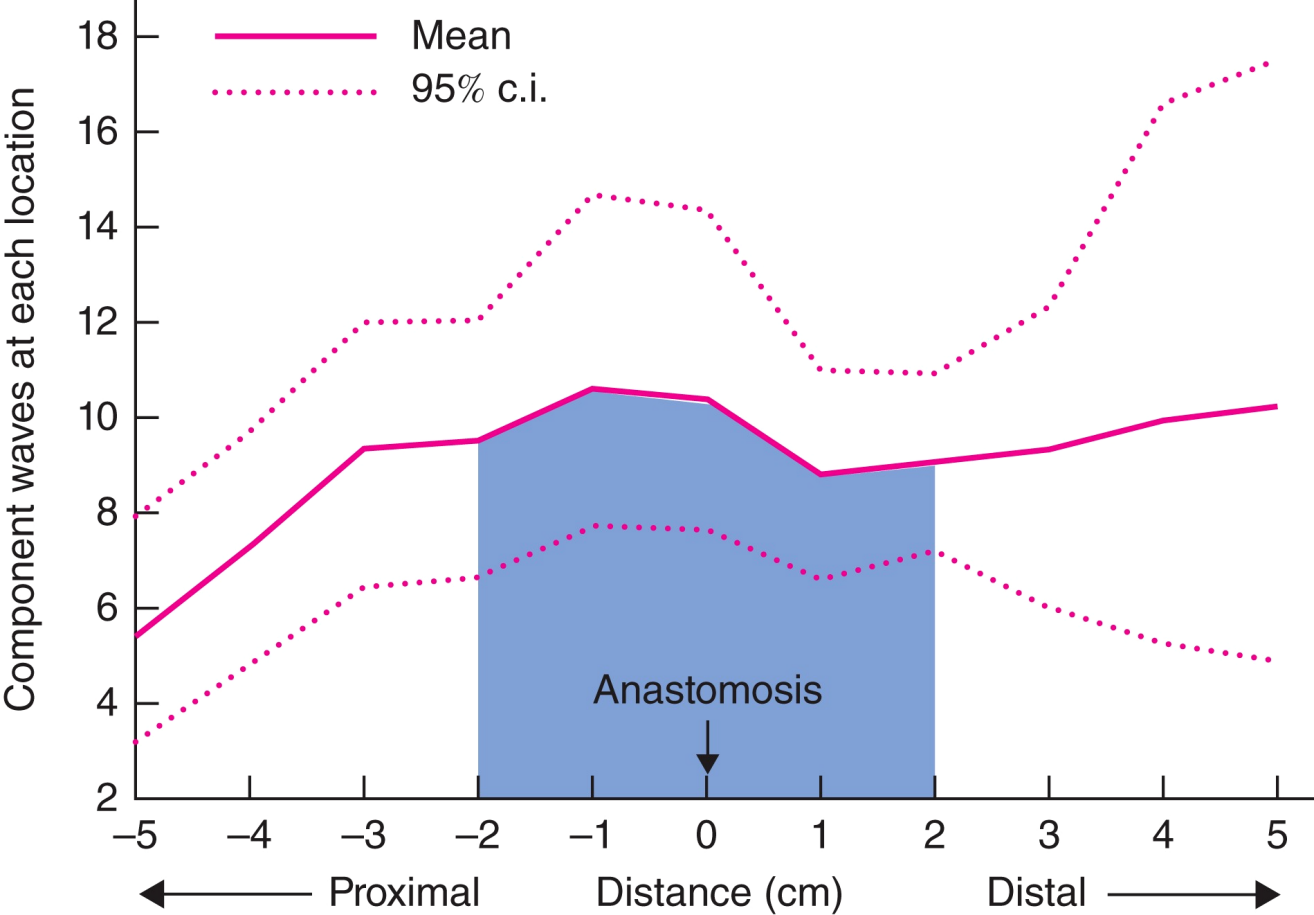
Adapted from Vather et al., 2016 with permission. Density analysis mapping of the number of propagating events occurring within 5 cm of the anastomosis. Some 47% of all propagating contractions occurred within 2 cm of the anastomosis (shaded area), with no drop-off at the site of the anastomosis.

Additionally, Vather et al. showed that in 9 out of 12 patients with “normal” bowel function post-anterior resection, propagating motor patterns (such as HAPCs and the CMP) traversed the anastomosis, with no drop-off at the site of the surgical scar (**Figure 4**, adapted with permission from Vather et al) (74). Return of trans-anastomotic propagation was hypothesised to have occurred through regeneration of neural tissues and/or ICC networks as seen in the small bowel (76, 77), and previous animal models (78–80), rather than be explained by locally mediated stretch responses as these studies were undertaken in prepared colons. This presumed trans-anastomotic ICC regrowth with restoration of ICC-mediated motility suggests that extrinsic neural denervation may have a more significant role in LARS than ICC loss (74). This is further supported by low-resolution manometry studies which show reduced propagating contractions to the neorectum and prolonged colonic transit time with long denervation compared to short denervation (81, 82). While limited by low resolution techniques these studies were able to identify a significant correlation between altered motility and faecal urgency and multiple evacuations (82).

HRCM evidence has suggested that the loss of rectosigmoid brake function as well as loss of long gastrointestinal feedback loops (such as the gastrocolic reflex, defecation, or sacral autonomic reflexes) may be plausible mechanisms underlying LARS. These hypotheses are further supported by other physiological modalities; Mochiki et al. performed a barostat study in 37 patients after low anterior resection, 17 with high stool frequencies and 20 with normal stool frequencies and found an increased gastrocolic reflex in patients after low anterior resection (83). While a 300 kcal meal did not induce contractions in healthy controls, patients with increased stool frequency after low anterior resection had relative hypermotility proximal to the anastomosis (in the colonic conduit) compared to those with normal stool frequency (83). As these studies were performed using barostat techniques, it could not be resolved what specific motor patterns were occurring in the colon of these subjects. Others have also shown spastic motility of the neorectum to be associated with urge incontinence after sphincter preserving surgery (81, 82, 84). In an anorectal physiology study, 23 patients with major LARS had a hyperactive postprandial response with a significant increase in postprandial neorectal pressure compared to patients without LARS (n = 9) after total mesorectal excision (85). More recently, colonic transit studies have demonstrated that patients with major LARS have significantly faster transit compared to those with minor or no LARS (26). Increased transit could be a consequence of either hypofunction of the rectosigmoid brake, or overactivity of proximal propulsive activity, further supporting the role of altered colonic motility in LARS.

**Figure 5:**
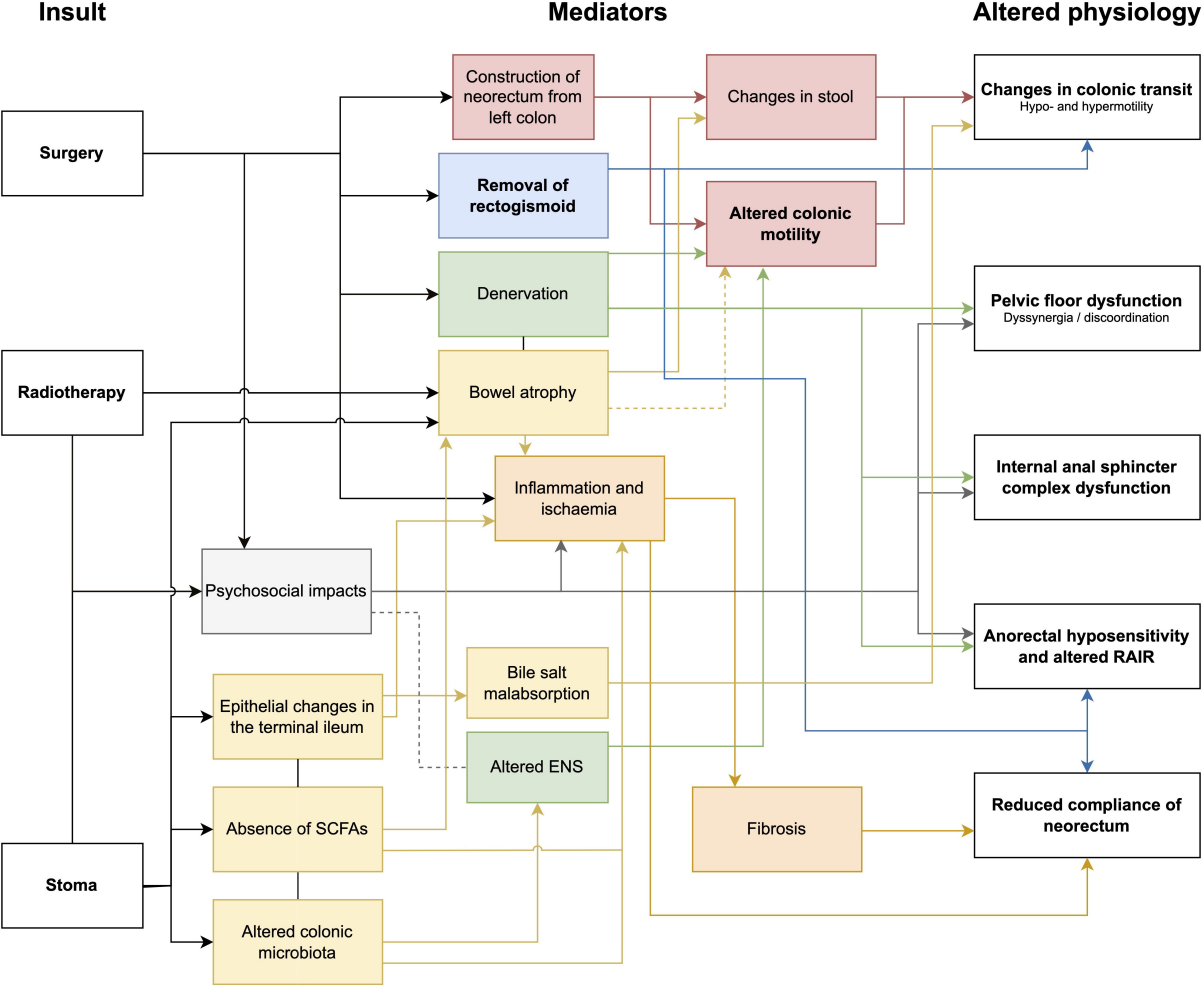
Flow diagram of mechanisms of injury as a consequence of surgery, radiotherapy and stoma formation. *SCFA, short chain fatty acids; ENS, enteric nervous system; rectoanal inhibitory reflex, RAIR*.

Colonic motility is likely an important contributor to the complex and multifactorial underlying pathophysiology of LARS. Other critical aetiological mechanisms during surgery relate to disruptions to intestinal continuity, denervation of the mobilised left colon, and injury to the anal sphincter complex. Construction of a neorectum with altered motility (often secondary to nerve injury) (85), and decreased functional capacity will also contribute to LARS. Other factors such as the consequences of faecal diversion and radiotherapy as well as postoperative medications, dietary and psychological factors are also important considerations that have been detailed thoroughly previously (16, 17). **Figure 5** shows the various impacts of surgery, radiotherapy and stoma formation, and the suggested mediators that contribute to the altered physiology underlying LARS.

## Risk factors and mechanisms for colonic dysfunction in LARS

While risk factors for LARS after anterior resection (such as anastomotic height, radiotherapy, temporary ileostomy) (11, 12) have been consistently established in the literature, the complex pathophysiological mechanisms underpinning these risk factors are still being defined. The following sections overviews postulated mechanisms for colonic dysmotility and dysfunction in LARS and are summarised in **Figures 2** and **5**.

### Correlation between anastomotic height and degree of colonic resection on LARS

Anastomotic height has been shown to be a consistent risk factors for poor functional outcome after rectal resection (10–12). The primary impact of a low anastomosis is reduced neorectal compliance and capacitance (17). Decreased neorectal volume has been associated with temporary neorectal irritability which occurs in response to an inability to accommodate neorectal filling (86). Lower anastomoses are also associated with increased risks of direct injury to the IAS or conjoint longitudinal ligament secondary to insertion of stapling devices and division of rectococcygeus and/or hiatal ligaments if required (17). Damage to the neural supply to the IAS is more likely with lower anastomoses, particularly when dissection reaches the posterolateral prostate in men (17). The pudendal nerve is typically spared in TME, with risk of damage usually only in the event of lateral pelvic lymph node dissection (17). Lower anastomoses have also been shown to be associated with a loss of the RAIR, but this often recovers over time (19).

Similarly, just as residual rectal length impacts neorectal capacity and compliance, the length of the remaining colon also likely has a role in LARS given the importance of distal colonic motility in bowel function. A greater length of functional descending and sigmoid colon left *in-situ* may allow greater compensation after rectosigmoid resection. The distal colon has important storage functions, which may reduce ability to delay defecation when these reserves are lost along with the rectal storage capacity (87). Resection of the rectosigmoid junction, removes the predominant site of origin for the CMP (53, 88), however CMP may also originate within the distal transverse and descending colon and the upper rectum (53, 88). It can therefore be expected that patients who retain greater lengths of residual colon may experience better bowel function after surgery, however this has not been studied to our knowledge.

### Impact of reconstruction technique

Alternative reconstruction methods such as side-to-end anastomoses (SEA) and colonic J pouches (CJP) are often used in an attempt to mitigate against bowel dysfunction associated with straight or end-to-end anastomoses (89). Functional outcomes have been thoroughly compared after various reconstructive techniques in numerous meta-analyses (90–97). When compared to straight anastomoses, CJP have been shown to be associated with lower stool frequency for up to 12-months, but no longer than 2 years (90, 92, 94, 95, 97, 98). SEA anastomoses have been associated with reduced incomplete defecation compared to CJP (97), but generally the two techniques have equivalent functional outcomes (90, 95, 96). In contrast to the vast literature on functional outcomes, there is sparse investigation of underlying physiological or mechanistic differences between the reconstruction techniques. Some studies found minimal differences between CJP and straight anastomoses (99), however there is some evidence that CJP are associated with greater maximum tolerable volume, greater maximum resting pressures, and improved pouch compliance (100–102), however these differences rarely persist beyond 2 years (103). Few small studies have assessed differences in functional outcomes or anorectal physiology between handsewn and stapled anastomoses. Generally, there is similar symptomatology (104) between the groups but one study reported higher incontinence scores in association with handsewn anastomoses despite no difference in anorectal physiology parameters (105, 106). Further investigation of differences in colonic or neorectal motility between reconstruction techniques may reveal important factors associated with functional outcomes.

### Impact of surgical denervation

Denervation of the colon and rectum contributes to the pathogenesis of LARS, and is likely a significant mediator of altered colonic motility after surgery (16). Surgical resection can cause neorectal dysmotility due to autonomic denervation (18) as resection of the rectum and associated blood vessels causes parasympathetic denervation and ‘high ligation’ at the origin of the inferior mesenteric artery (IMA) denervates accompanying sympathetic nerve fibres (81, 84, 107, 108). Depending on the surgical technique employed, the colonic conduit upstream of the resection will therefore be denervated to a variable extent (81). Koda et al. performed low resolution intraluminal pressure measurements and transit studies in 67 individuals after low or ultralow anterior resection and found intraoperative denervation of the remnant colon significantly disrupted motility (81). When these outcomes were compared for patients who had a long denervated segment used to create the neorectal conduit (i.e. high ligation with the IMA taken at its origin or the ascending fibres compromised during lymph node dissection) vs. patients who had a short denervated segment (the superior rectal artery divided at its origin) there was a tendency towards worse evacuatory outcomes in the long denervation group (81). In particular, IMA ligation resulted in fewer propagating contractions, but increased non-propagating minor contractions. Accordingly, colonic transit was relatively prolonged in those that had high IMA ligation (81). Koda et al. also associated resultant colonic dysmotility with multiple evacuations, urgency and soiling (81).

These findings in post-surgical cohorts are consistent with animal studies evaluating pathophysiological mechanisms. Research in canine and murine models has shown that denervation of the sympathetic supply to the colon *via* either surgical or chemical neurectomy of the IMA pedicle increases rectal motility and colonic transit, while denervation of the parasympathetic supply has the opposite effect (84, 109–111). This effect of denervation, whereby distal colonic activity increased with corresponding increase in stool frequency, was independent of colonic transection in this study of rats (84, 110). Using strain gauge transducers implanted on the serosal surface of the descending colon of male rats, Lee et al. confirmed denervation of the left colon was associated with increased colonic motility, likely mediated through inhibitory alpha-sympathetic pathways (110). Non-propagatory contractile activity has been associated with faecal soiling, urgency, multiple evacuations and dissatisfaction with defecatory function (81, 82). However, these findings are not universal, as changes in motility can be associated with heterogenous changes in transit as evidenced by other studies demonstrating shorter colonic transit times after anterior resection (26, 112). Additionally, the use of low-resolution techniques in such studies, with pressure sensors spaced up to 20 cm apart (81, 82), limits the interpretation of these results because total colonic activity may be misinterpreted and underestimated (113). It should be noted there remains ongoing debate on the influence of the level of IMA ligation on functional outcomes, with a potentially greater impact on genitourinary function (114, 115).

### Impact of radiotherapy

Pelvic radiotherapy is an important aspect of rectal cancer management, however frequently results in persistent gastrointestinal symptoms substantially impacting patients’ quality of life (116, 117). Primary mechanisms of radiotherapy-induced symptoms include dysfunction of neural, enzymatic, and muscular function, acute inflammation, chronic cytokine activation, chronic colonic ischaemia, and fibrosis of the colonic wall, stroma and mesentery (117). However, inflammatory histopathological abnormalities correlate poorly with symptoms (118, 119). Colonic dysmotility is often seen in inflammatory bowel diseases (120, 121), as such it is conceivable that an inflammatory insult to the colon through radiotherapy may also contribute to symptoms associated with ‘pelvic radiation disease’. The relative contributions of these different mechanisms from multiple physiologic insults in patients undergoing rectal cancer treatment remain difficult to separate.

As outlined above, post-surgical neural disruption can be a significant contributor to LARS aetiology. Similarly, neuropathy can also arise secondary to radiotherapy, largely through the radiation induced fibrosis, atrophy, and ulceration to neural tissues (122). Bondeven et al. showed that functional benefits of a larger rectal remnant were abolished by neoadjuvant radiotherapy through disruption of afferent signalling, potential changes in cortical processing of neorectal signals, and brain-gut axis dysregulation (123). Both surgical and radiotherapy insults also disrupt the autonomic coordination of the recto-anal inhibitory reflex (124, 125). This is congruent with neoadjuvant radiotherapy being a strong and consistently identified risk factor for LARS in cross-sectional and longitudinal studies (12, 126).

The gut is particularly radiosensitive due to high mucosal turnover (127, 128), which results in elevated inflammatory cytokines (such as IL-2, IL-6, IL-8, and particularly IL-1β) (129). These pro-inflammatory sequelae can disrupt the gastrointestinal milieu causing changes in the gut microbiome composition, resulting in symptoms like diarrhoea (130). Radiotherapy can cause local gastrointestinal dysbiosis which in turn can promote colitis symptoms (131). Long term radiation changes to rectal tissue not only promotes fibrosis but ischaemic changes *via* obliterative endarteritis which can facilitate necrosis or ulceration (132). Mechanisms for radiation-related intestinal inflammation are under active investigation, particularly the role of inflammatory cell extravasation *via* leukocyte-endothelium adhesion, and there is more to discover on how these factors impact colonic physiology and LARS (133). Dietary fibre has anti-inflammatory properties which protect the colonic mucosa, reduce carcinogenesis, and has been shown to ameliorate the effects of LARS (134). The beneficial effects of short-chain fatty acids are thought to act through HDAC inhibition and GPR activation as summarised in a recent review (135).

### Impact of ileostomy

Diverting ileostomies are commonly used to mitigate the harms of an anastomotic leak, particularly in the setting of low colorectal anastomoses. One systematic review of 8 studies reported an ileostomy rate of 61.2% (range 29% - 100%) among patients that had LAR for rectal cancer with colorectal or coloanal anastomosis (136). However, while protecting the anastomosis, it is important to consider the impacts of faecal diversion. ​​Ileostomy formation has been consistently identified to be a risk factor for postoperative bowel dysfunction (12, 137–141); a relationship that persists >18 months after reversal of ileostomy (12). It may be due to colonocyte malnutrition with resulting bowel atrophy and/or inflammatory sequelae in the distal bowel, nutritional deficit affecting the enteral neural plexi (134, 142), colonic dysbiosis, or epithelial changes in the terminal ileum itself. Loss of intra-luminal nutrition in the defunctioned bowel, particularly short-chain fatty acids like acetate, propionate, and butyrate can impair colonic homeostasis (134). The anti-inflammatory effects of these substances, particularly dietary fibre, which protect the colonic mucosa are lost. A defunctioning ileostomy can also cause intestinal dysbiosis which in turn causes mucosal atrophy (142), reducing absorptive capacity, increasing the risk of postoperative complications, and exacerbating homeostatic derangements. The potential to identify mediators (such as dietary fibre) which may mitigate some of the effects may introduce further therapeutic targets.

## Future directions

LARS pathophysiology is receiving increased attention and this review highlights the importance of a standardised definition of LARS to guide consistent clinical investigation. Given the varying symptoms experienced, numerous mechanisms are likely to contribute to LARS, with specific symptom experiences potentially explained by overlapping factors. The role of colonic motility has been a relatively underappreciated contributor. Further work is required to better correlate symptoms of LARS with profiles of colonic dysmotility and such advances may direct targeted therapies, including SNM through its modulation of colonic motility (41). Additionally, it is of interest to see how motility profiles change over time, particularly beyond 18-months when function tends to improve (12). As highlighted in this review, colonic motility assessments should be included in a comprehensive physiological assessment of patients with LARS.

The highly invasive nature of colonic manometry has limited the study of colonic motility, often resulting in small study cohorts. Emerging technologies that enable non-invasive detection of colonic myoelectrical activity through skin-surface electrical recordings have demonstrated the ability to detect and quantify meal responses (143). These approaches offer a promising avenue to detect changes in motility profiles, including in the rectosigmoid brake, in a scalable format. Given the heterogeneity of LARS, widely accessible, non-invasive investigations will be required to better understand the causes of dysfunction and characterise individual phenotypes of LARS. Ultimately, it is hoped that accurate phenotyping may facilitate more mechanistically targeted and therefore efficacious therapies for patients with LARS. This is a vital step towards improving quality of life in rectal cancer survivors.

## Conclusions

Altered distal colonic motility is an important contributor to the pathophysiology of LARS and may offer an actionable biomarker for targeted therapies. Further work is required to enable accessible and accurate assessment of colonic motility, to guide understanding of LARS aetiology and to facilitate physiologically congruent management strategies for these patients.

## Author contributions

IB, GO’G, and CK, provided supervision. CV, CW, IB, GO’G, and CK wrote the paper and approved the final version for publication.

## Funding

This work was supported by the New Zealand Health Research Council and the Royal Australasian College of Surgeons’ John Mitchell Crouch Fellowship.

## Acknowledgments

We would like to thank Patrick Lane for their assistance with the medical illustrations.

## Conflict of interest

GO’G, IB, and CK are members of The University of Auckland Spin-out companies: The Insides Company Ltd (GO’G, IB, CK), and Alimetry Ltd (GO’G).

The remaining authors declare that the research was conducted in the absence of any commercial or financial relationships that could be construed as a potential conflict of interest.

## Publisher’s note

All claims expressed in this article are solely those of the authors and do not necessarily represent those of their affiliated organizations, or those of the publisher, the editors and the reviewers. Any product that may be evaluated in this article, or claim that may be made by its manufacturer, is not guaranteed or endorsed by the publisher.

## References

[1] Dekker E Tanis PJ Vleugels JLA Kasi PM Wallace MB. Colorectal cancer. Lancet (2019) 394:1467–80. doi: 10.1016/S0140-6736(19)32319-0 31631858

[2] Brännström F Bjerregaard JK Winbladh A Nilbert M Revhaug A Wagenius G Mörner M. Multidisciplinary team conferences promote treatment according to guidelines in rectal cancer. Acta Oncol (2015) 54:447–53. doi: 10.3109/0284186X.2014.952387 25291075

[3] van Gijn W Marijnen CAM Nagtegaal ID Kranenbarg EM-K Putter H Wiggers T. Preoperative radiotherapy combined with total mesorectal excision for resectable rectal cancer: 12-year follow-up of the multicentre, randomised controlled TME trial. Lancet Oncol vol (2011) 12:575–82. doi: 10.1016/S1470-2045(11)70097-3 21596621

[4] Heald RJ Ryall RD. Recurrence and survival after total mesorectal excision for rectal cancer. Lancet (1986) 1:1479–82. doi: 10.1016/S0140-6736(86)91510-2 2425199

[5] Kupsch J Kuhn M Matzel KE Zimmer J Radulova-Mauersberger O Sims A. To what extent is the low anterior resection syndrome (LARS) associated with quality of life as measured using the EORTC C30 and CR38 quality of life questionnaires? Int J Colorectal Dis (2019) 34:747–62. doi: 10.1007/s00384-019-03249-7 30721417

[6] Kong JC Soucisse M Michael M Tie J Ngan SY Leong T. Total neoadjuvant therapy in locally advanced rectal cancer: A systematic review and metaanalysis of oncological and operative outcomes. Ann Surg Oncol (2021) 28:7476–86. doi: 10.1245/s10434-021-09837-8 33891203

[7] CollaborativeR Zaborowski AM Abdile A Adamina M Aigner F d’Allens L. Characteristics of early-onset vs late-onset colorectal cancer: A review. JAMA Surg (2021) 156:865–74. doi: 10.1001/jamasurg.2021.2380 34190968

[8] Araghi M Soerjomataram I Jenkins M Brierley J Morris E Bray F. Global trends in colorectal cancer mortality: projections to the year 2035. Int J Cancer (2019) 144:2992–3000. doi: 10.1002/ijc.32055 30536395

[9] Keane C Fearnhead NS Bordeianou LG Christensen P Basany EE Laurberg S. International consensus definition of low anterior resection syndrome. Dis Colon Rectum (2020) 63:274–84. doi: 10.1097/DCR.0000000000001583 PMC7034376 32032141

[10] Croese AD Lonie JM Trollope AF Vangaveti VN Ho Y-H. A meta-analysis of the prevalence of low anterior resection syndrome and systematic review of risk factors. Int J Surg vol (2018) 56:234–41. doi: 10.1016/j.ijsu.2018.06.031 29936195

[11] Sun R Dai Z Zhang Y Lu J Zhang Y Xiao Y. The incidence and risk factors of low anterior resection syndrome (LARS) after sphincter-preserving surgery of rectal cancer: a systematic review and meta-analysis. Support Care Cancer (2021); 29(12):7249–58. doi: 10.1007/s00520-021-06326-2 34296335

[12] Varghese C Wells CI O’Grady G Christensen P Bissett IP Keane C. The longitudinal course of low-anterior resection syndrome: An individual patient meta-analysis. Ann Surg (2022); 276(1):46–54. doi: 10.1097/SLA.0000000000005423 35185131

[13] Juul T Ahlberg M Biondo S Espin E Jimenez LM Matzel KE. Low anterior resection syndrome and quality of life: an international multicenter study. Dis Colon Rectum (2014) 57:585–91. doi: 10.1097/DCR.0000000000000116 24819098

[14] van der Valk MJM Hilling DE Bastiaannet E Meershoek-Klein Kranenbarg E Beets GL Figueiredo NL. Long-term outcomes of clinical complete responders after neoadjuvant treatment for rectal cancer in the international watch & wait database (IWWD): an international multicentre registry study. Lancet (2018) 391:2537–45. doi: 10.1016/S0140-6736(18)31078-X 29976470

[15] Smith JJ Strombom P Chow OS Roxburgh CS Lynn P Eaton A. Assessment of a watch-and-Wait strategy for rectal cancer in patients with a complete response after neoadjuvant therapy. JAMA Oncol (2019) 5:e185896. doi: 10.1001/jamaoncol.2018.5896 30629084 PMC6459120

[16] Christensen P Im Baeten C Espín-Basany E Martellucci J Nugent KP Zerbib F. Management guidelines for low anterior resection syndrome - the MANUEL project. Colorectal Dis (2021) 23:461–75. doi: 10.1111/codi.15517 PMC7986060 33411977

[17] Koda K Yamazaki M Shuto K Kosugi C Mori M Narushima K. Etiology and management of low anterior resection syndrome based on the normal defecation mechanism. Surg Today (2019) 49:803–8. doi: 10.1007/s00595-019-01795-9 30937634

[18] Catchpole BN. Motor pattern of the left colon before and after surgery for rectal cancer: possible implications in other disorders. Gut (1988) 29:624–30. doi: 10.1136/gut.29.5.624 PMC1433669 3396950

[19] Lee SJ Park YS. Serial evaluation of anorectal function following low anterior resection of the rectum. Int J Colorectal Dis (1998) 13:241–6. doi: 10.1007/s003840050169 9870169

[20] Wong RKS Tandan V De Silva S Figueredo A. Pre-operative radiotherapy and curative surgery for the management of localized rectal carcinoma. Cochrane Database Syst Rev (2007) 2:CD002102. doi: 10.1002/14651858.CD002102.pub2 17443515

[21] Nesbakken A Nygaard K Lunde OC. Mesorectal excision for rectal cancer: functional outcome after low anterior resection and colorectal anastomosis without a reservoir. Colorectal Dis (2002) 4:172–6. doi: 10.1046/j.1463-1318.2002.00305.x 12780611

[22] Kakodkar R Gupta S Nundy S. Low anterior resection with total mesorectal excision for rectal cancer: functional assessment and factors affecting outcome. Colorectal Dis (2006) 8:650–6. doi: 10.1111/j.1463-1318.2006.00992.x 16970574

[23] Bakx R Doeksen A Slors JFM Bemelman WA van Lanschot JJB Boeckxstaens GEE. Neorectal irritability after short-term preoperative radiotherapy and surgical resection for rectal cancer. Am J Gastroenterol (2009) 104:133–41. doi: 10.1038/ajg.2008.2 19098861

[24] Williamson ME Lewis WG Finan PJ Miller AS Holdsworth PJ Johnston D. Recovery of physiologic and clinical function after low anterior resection of the rectum for carcinoma: myth or reality? Dis Colon Rectum (1995) 38:411–8. doi: 10.1007/BF02054232 7720451

[25] Keane C Paskaranandavadivel N Vather R Rowbotham D Arkwright J Dinning P. Altered colonic motility is associated with low anterior resection syndrome. Colorectal Dis (2021) 23:415–23. doi: 10.1111/codi.15465 33253472

[26] Ng K-S Russo R Gladman MA. Colonic transit in patients after anterior resection: prospective, comparative study using single-photon emission CT/CT scintigraphy. Br J Surg (2020) 107:567–79. doi: 10.1002/bjs.11471 32154585

[27] Keane C Wells C O’Grady G Bissett IP. Defining low anterior resection syndrome: a systematic review of the literature. Colorectal Dis (2017) 19:713–22. doi: 10.1111/codi.13767 28612460

[28] Emmertsen KJ Laurberg S. Low anterior resection syndrome score: development and validation of a symptom-based scoring system for bowel dysfunction after low anterior resection for rectal cancer. Ann Surg (2012) 255:922–8. doi: 10.1097/SLA.0b013e31824f1c21 22504191

[29] Ribas Y Aguilar F Jovell-Fernandez E Cayetano L Navarro-Luna A Munoz-Duyos A.. Clinical application of the LARS score: results from a pilot study. Int J Colorectal Dis (2017) 32:409–18. doi: 10.1007/s00384-016-2690-7 27796496

[30] Harji D Fernandez B Boissieras L Berger A Capdepont M Zerbib F. A novel bowel rehabilitation programme after total mesorectal excision for rectal cancer: the BOREAL pilot study. Colorectal Dis (2021); 23(10):2619–2626. doi: 10.1111/codi.15812 34264005

[31] Martellucci J. Low anterior resection syndrome. Dis Colon Rectum (2016) 59:79–82. doi: 10.1097/DCR.0000000000000495 26651116

[32] Chen TY-T Emmertsen KJ. Bowel dysfunction after rectal cancer treatment: A study comparing the specialist’s versus patient’s perspective. BMJ Open (2014) 4:e003374. doi: 10.1136/bmjopen-2013-003374 PMC3902194 24448844

[33] Mercado-Perez A Beyder A. Gut feelings: mechanosensing in the gastrointestinal tract. Nat Rev Gastroenterol Hepatol (2022) 19:283–96. doi: 10.1038/s41575-021-00561-y PMC9059832 35022607

[34] Huizinga JD Hussain A Chen J-H. Interstitial cells of cajal and human colon motility in health and disease. Am J Physiol Gastrointest Liver Physiol (2021) 321:G552–75. doi: 10.1152/ajpgi.00264.2021 34612070

[35] Lee H-T Hennig GW Park KJ Bayguinov PO Ward SM Sanders KM. Heterogeneities in ICC Ca2+ activity within canine large intestine. Gastroenterology (2009) 136:2226–36. doi: 10.1053/j.gastro.2009.02.060 PMC4802971 19268670

[36] Wells CI O’Grady G. *Interstitial Cells Cajal*. Encyclopedia of Gastroenterology, 2nd Edition. Interstitial Cells of Cajal (2020); 267–74. doi: 10.1016/B978-0-12-801238-3.65887-7

[37] Komuro T. Structure and organization of interstitial cells of cajal in the gastrointestinal tract. J Physiol (2006) 576:653–8. doi: 10.1113/jphysiol.2006.116624 PMC1890414 16916909

[38] Gillis RA Dias Souza J Hicks KA Mangel AW Pagani FD Hamilton BL. Inhibitory control of proximal colonic motility by the sympathetic nervous system. Am J Physiology-Gastrointestinal Liver Physiol (1987) 253:G531–9. doi: 10.1152/ajpgi.1987.253.4.G531 2889367

[39] Palit S Lunniss PJ Scott SM. The physiology of human defecation. Dig Dis Sci (2012) 57:1445–64. doi: 10.1007/s10620-012-2071-1 22367113

[40] Lin AY Dinning PG Milne T Bissett IP O’Grady G. The “rectosigmoid brake”: Review of an emerging neuromodulation target for colorectal functional disorders. Clin Exp Pharmacol Physiol (2017) 44:719–28. doi: 10.1111/1440-1681.12760 28419527

[41] Lin AY Varghese C Paskaranandavadivel N Seo S Du P Dinning P. Faecal incontinence is associated with an impaired rectosigmoid brake and improved by sacral neuromodulation. Colorectal Dis. (2022); 00: 1–11. doi: 10.1111/codi.16249 PMC10084032 35793162

[42] Rao SS Welcher K. Periodic rectal motor activity: the intrinsic colonic gatekeeper? Am J Gastroenterol (1996) 91:890–7. 8633577

[43] deSouza NM Williams AD Wilson HJ Gilderdale DJ Coutts GA Black CM. Fecal incontinence in scleroderma: assessment of the anal sphincter with thin-section endoanal MR imaging. Radiology (1998) 208:529–35. doi: 10.1148/radiology.208.2.9680588 9680588

[44] Heitmann PT Vollebregt PF Knowles CH Lunniss PJ Dinning PG Scott SM. Understanding the physiology of human defaecation and disorders of continence and evacuation. Nat Rev Gastroenterol Hepatol (2021) 18:751–69. doi: 10.1038/s41575-021-00487-5 34373626

[45] Galapeaux EA Templeton RD. The influence of filling the stomach on the colon motility and defecation in the dog. Am J Med Sci (1938) 195:230–3. doi: 10.1097/00000441-193802000-00012

[46] Hines LE Lueth HC Ivy AC. MOTILITY OF THE RECTUM IN NORMAL AND IN CONSTIPATED SUBJECTS. Arch Intern Med (1929) 44:147–52. doi: 10.1001/archinte.1929.00140010150012

[47] Almy TP Kern F Jr. Tulin M. Alterations in colonic function in man under stress; experimental production of sigmoid spasm in healthy persons. Gastroenterology (1949) 12:425–36. doi: 10.1016/S0016-5085(49)80125-9 18116018

[48] Ballantyne GH. Rectosigmoid sphincter of O’Beirne. Dis Colon Rectum (1986) 29:525–31. doi: 10.1007/BF02562612 3525042

[49] Narducci F Bassotti G Gaburri M Morelli A. Twenty four hour manometric recording of colonic motor activity in healthy man. Gut (1987) 28:17–25. doi: 10.1136/gut.28.1.17 PMC1432711 3817580

[50] Bharucha AE. High amplitude propagated contractions. Neurogastroenterol Motil (2012) 24:977–82. doi: 10.1111/nmo.12019 PMC3471560 23057554

[51] Bassotti G Betti C Fusaro C Morelli A. Colonic high-amplitude propagated contractions (mass movements): repeated 24-h manometric studies in healthy volunteers. Neurogastroenterol Motil (2008) 4:187–91. doi: 10.1111/j.1365-2982.1992.tb00160.x

[52] Milkova N Parsons SP Ratcliffe E Huizinga JD Chen J. On the nature of high-amplitude propagating pressure waves in the human colon. Am J Physiol Gastrointest Liver Physiol (2020); 318(4):646–60. doi: 10.1152/ajpgi.00386.2019 PMC7191456 32068445

[53] Dinning PG Wiklendt L Maslen L Gibbins I Patton V Arkwright JW. Quantification of *in vivo*colonic motor patterns in healthy humans before and after a meal revealed by high-resolution fiber-optic manometry. Neurogastroenterol Motil (2014) 26:1443–57. doi: 10.1111/nmo.12408 PMC4438670 25131177

[54] Lin AY Du P Dinning PG Arkwright JW Kamp JP Cheng LK. High-resolution anatomic correlation of cyclic motor patterns in the human colon: Evidence of a rectosigmoid brake. Am J Physiol - Gastrointestinal Liver Physiol (2017) 312:G508–15. doi: 10.1152/ajpgi.00021.2017 PMC5451563 28336544

[55] Chen J-H Nirmalathasan S Pervez M Milkova N Huizinga JD. The sphincter of O’Beirne – part 1: Study of 18 normal subjects. Digestive Dis Sci (2021) 66:3516–28. doi: 10.1007/s10620-020-06657-w 33462748

[56] Heitmann PT Mohd Rosli R Maslen L Wiklendt L Kumar R Omari TI. High-resolution impedance manometry characterizes the functional role of distal colonic motility in gas transit. Neurogastroenterol Motil (2022) 34:e14178. doi: 10.1111/nmo.14178 34076936

[57] Lin AY Varghese C Du P Wells CI Paskaranandavadivel N Gharibans AA. Intraoperative serosal extracellular mapping of the human distal colon: a feasibility study. Biomed Eng Online (2021) 20:105. doi: 10.1186/s12938-021-00944-x PMC8520224 34656127

[58] Cook IJ Brookes SJ Dinning PG. Colonic motor and sensory function and dysfunction. Sleisenger Fordtran’s Gastrointestinal Liver Dis (2010), 1659–1674.e1. doi: 10.1016/b978-1-4160-6189-2.00098-6

[59] Aaronson MJ Freed MM Burakoff R. Colonic myoelectric activity in persons with spinal cord injury. Dig Dis Sci (1985) 30:295–300. doi: 10.1007/BF01403836 3979236

[60] Fajardo NR Pasiliao R-V Modeste-Duncan R Creasey G Bauman WA Korsten MA. Decreased colonic motility in persons with chronic spinal cord injury. Am J Gastroenterol (2003) 98:128–34. doi: 10.1111/j.1572-0241.2003.07157.x 12526948

[61] Battle WM Snape WJ Jr Wright S Sullivan MA Cohen S Meyers A. Abnormal colonic motility in progressive systemic sclerosis. Ann Intern Med (1981) 94:749–52. doi: 10.7326/0003-4819-94-6-749 7235416

[62] Battle WM Snape WJ Jr. Alavi A Cohen S Braunstein S. Colonic dysfunction in diabetes mellitus. Gastroenterology (1980) 79:1217–21. doi: 10.1016/0016-5085(80)90916-6 7439629

[63] Boyle DJ Knowles CH Lunniss PJ Scott SM Williams NS Gill KA. Efficacy of sacral nerve stimulation for fecal incontinence in patients with anal sphincter defects. Dis Colon Rectum (2009) 52:1234–9. doi: 10.1007/DCR.0b013e31819f7400 19571698

[64] Ratto C Litta F Parello A Donisi L De Simone V Zaccone G. Sacral nerve stimulation in faecal incontinence associated with an anal sphincter lesion: a systematic review. Colorectal Dis (2012) 14:e297–304. doi: 10.1111/j.1463-1318.2012.03003.x 22356165

[65] Patton V Wiklendt L Arkwright JW Lubowski DZ Dinning PG. The effect of sacral nerve stimulation on distal colonic motility in patients with faecal incontinence. Br J Surg (2013) 100:959–68. doi: 10.1002/bjs.9114 23536312

[66] Michelsen HB Christensen P Krogh K Rosenkilde M Buntzen S Theil J. Sacral nerve stimulation for faecal incontinence alters colorectal transport. Br J Surg (2008) 95:779–84. doi: 10.1002/bjs.6083 18412293

[67] Y. H Koh CE. Sacral nerve stimulation for bowel dysfunction following low anterior resection: a systematic review and meta-analysis. Colorectal Dis (2019) 21:1240–8. doi: 10.1111/codi.14690 31081580

[68] Chen J-H Collins SM Milkova N Pervez M Nirmalathasan S Tan W. The sphincter of O’Beirne-part 2: Report of a case of chronic constipation with autonomous dyssynergia. Dig Dis Sci (2021) 66:3529–41. doi: 10.1007/s10620-020-06723-3 33462747

[69] Bassotti G Chistolini F Battaglia E Chiarioni G Nzepa FS Dughera L. Are colonic regular contractile frequency patterns in slow transit constipation a relevant pathophysiological phenomenon? Dig Liver Dis (2003) 35:552–6. doi: 10.1016/S1590-8658(03)00271-8 14567459

[70] Rao SS Sadeghi P Batterson K Beaty J. Altered periodic rectal motor activity: a mechanism for slow transit constipation. Neurogastroenterol Motil (2001) 13:591–8. doi: 10.1046/j.1365-2982.2001.00292.x 11903920

[71] Vather R O’Grady G Lin AY Du P Wells CI Rowbotham D. Hyperactive cyclic motor activity in the distal colon after colonic surgery as defined by high-resolution colonic manometry. Br J Surg (2018) 105:907–17. doi: 10.1002/bjs.10808 PMC7938810 29656582

[72] Seo SHB Bissett I O’Grady G. Variable gut function recovery after right vs left colectomy may be due to rectosigmoid hyperactivity. Front Physiol (2021) 12:91. doi: 10.3389/fphys.2021.635167 PMC7940204 33708140

[73] Wells CI Penfold JA Paskaranandavadivel N Rowbotham D Du P Seo S. Hyperactive distal colonic motility and recovery patterns following right colectomy: a high-resolution manometry study. Dis Colon Rectum (2022) doi: 10.1097/DCR.00000000000023 35499821

[74] Vather R O’Grady G Arkwright JW Rowbotham DS Cheng LK Dinning PG. Restoration of normal colonic motor patterns and meal responses after distal colorectal resection. Br J Surg (2016) 103:451–61. doi: 10.1002/bjs.10074 26780492

[75] Asnong A Tack J Devoogdt N De Groef A Geraerts I D’Hoore A. Exploring the pathophysiology of LARS after low anterior resection for rectal cancer with high-resolution colon manometry. Neurogastroenterol Motil (2022) e14432. doi: 10.1111/nmo.14432 35866548

[76] Galligan JJ Furness JB Costa M. Migration of the myoelectric complex after interruption of the myenteric plexus: intestinal transection and regeneration of enteric nerves in the guinea pig. Gastroenterology (1989) 97:1135–46. doi: 10.1016/0016-5085(89)91683-1 2571545

[77] Wang TH-H Angeli TR Beban G Du P Bianco F Gibbons SJ. Slow-wave coupling across a gastroduodenal anastomosis as a mechanism for postsurgical gastric dysfunction: evidence for a “gastrointestinal aberrant pathway”. Am J Physiology-Gastrointestinal Liver Physiol (2019) 317:G141–6. doi: 10.1152/ajpgi.00002.2019 PMC6734376 31169993

[78] Horgan AF Molloy RG Coulter J Sheehan M Kirwan WO. Nerve regeneration across colorectal anastomoses after low anterior resection in a canine model. Int J Colorectal Dis (1993) 8:167–9. doi: 10.1007/BF00341192 8245674

[79] Brookes SJ Lam TC Lubowski DZ Costa M King DW. Regeneration of nerve fibres across a colonic anastomosis in the guinea-pig. J Gastroenterol Hepatol (1996) 11:325–34. doi: 10.1111/j.1440-1746.1996.tb01379.x 8713698

[80] Hanani M Ledder O Yutkin V Abu-Dalu R Huang T-Y Härtig W. Regeneration of myenteric plexus in the mouse colon after experimental denervation with benzalkonium chloride. J Comp Neurol (2003) 462:315–27. doi: 10.1002/cne.10721 12794735

[81] Koda K Saito N Seike K Shimizu K Kosugi C Miyazaki M.. Denervation of the neorectum as a potential cause of defecatory disorder following low anterior resection for rectal cancer. Dis Colon Rectum (2005) 48:210–7. doi: 10.1007/s10350-004-0814-6 15711859

[82] Iizuka I Koda K Seike K Shimizu K Takami Y Fukuda H. Defecatory malfunction caused by motility disorder of the neorectum after anterior resection for rectal cancer. Am J Surg (2004) 188:176–80. doi: 10.1016/j.amjsurg.2003.12.064 15249246

[83] Mochiki E Nakabayashi T Suzuki H Haga N Fujita K Asao T. Barostat examination of proximal site of the anastomosis in patients with rectal cancer after low anterior resection. World J Surg (2001) 25:1377–82. doi: 10.1007/s00268-001-0144-y 11760737

[84] Shimizu K Koda K Kase Y Satoh K Seike K Nishimura M. Induction and recovery of colonic motility/defecatory disorders after extrinsic denervation of the colon and rectum in rats. Surgery (2006) 139:395–406. doi: 10.1016/j.surg.2005.08.018 16546505

[85] Emmertsen KJ Bregendahl S Fassov J Krogh K Laurberg S. A hyperactive postprandial response in the neorectum–the clue to low anterior resection syndrome after total mesorectal excision surgery? Colorectal Dis (2013) 15:e599–606. doi: 10.1111/codi.12360 23869468

[86] Pucciani F. A review on functional results of sphincter-saving surgery for rectal cancer: the anterior resection syndrome. Updates Surg (2013) 65:257–63. doi: 10.1007/s13304-013-0220-5 23754496

[87] Hammer J Pruckmayer M Bergmann H Kletter K Gangl A. The distal colon provides reserve storage capacity during colonic fluid overload. Gut (1997) 41:658–63. doi: 10.1136/gut.41.5.658 PMC1891583 9414974

[88] Pervez M Ratcliffe E Parsons SP Chen J-H Huizinga JD. The cyclic motor patterns in the human colon. Neurogastroenterol Motil (2020) 32:e13807. doi: 10.1111/nmo.13807 32124528

[89] Bryant CLC Lunniss PJ Knowles CH Thaha MA Chan CLH. Anterior resection syndrome. Lancet Oncol (2012) 13:e403–8. doi: 10.1016/S1470-2045(12)70236-X 22935240

[90] Brown CJ Fenech DS McLeod RS. Reconstructive techniques after rectal resection for rectal cancer. Cochrane Database Syst Rev (2008) 2010:CD006040. doi: 10.1002/14651858.CD006040.pub2 PMC8911547 18425933

[91] Liao C Gao F Cao Y Tan A Li X Wu D. Meta-analysis of the colon J-pouch vs transverse coloplasty pouch after anterior resection for rectal cancer. Colorectal Dis (2010) 12:624–31. doi: 10.1111/j.1463-1318.2009.01964.x 19555386

[92] Heriot AG Tekkis PP Constantinides V Paraskevas P Nicholls RJ Darzi A. Meta-analysis of colonic reservoirs versus straight coloanal anastomosis after anterior resection. Br J Surg (2006) 93:19–32. doi: 10.1002/bjs.5188 16273532

[93] Temple LKF McLeod RS. A meta-analysis comparing functional outcome following straight coloanal anastomosis versus a colonic J pouch. Semin Colon Rectal Surg (2002) 13:62–6. doi: 10.1053/scrs.2002.31446

[94] Murphy J Hammond TM Knowles CH Scott SM Lunniss PJ Williams NS. Does anastomotic technique influence anorectal function after sphincter-saving rectal cancer resection? a systematic review of evidence from randomized trials. J Am Coll Surg (2007) 204:673–80. doi: 10.1016/j.jamcollsurg.2007.01.008 17382228

[95] Hüttner FJ Tenckhoff S Jensen K Uhlmann L Kulu Y Büchler MW. Meta-analysis of reconstruction techniques after low anterior resection for rectal cancer. Br J Surg (2015) 102:735–45. doi: 10.1002/bjs.9782 25833333

[96] Wang Z. Colonic J-pouch versus side-to-end anastomosis for rectal cancer: a systematic review and meta-analysis of randomized controlled trials. BMC Surg (2021) 21:331. doi: 10.1186/s12893-021-01313-0 PMC8379825 34419022

[97] Hou S Wang Q Zhao S Liu F Guo P Ye Y. Safety and efficacy of side-to-end anastomosis versus colonic J-pouch anastomosis in sphincter-preserving resections: an updated meta-analysis of randomized controlled trials. World J Surg Oncol (2021) 19:130. doi: 10.1186/s12957-021-02243-0 PMC8061176 33882952

[98] Zaman S Mohamedahmed AYY Ayeni AA Peterknecht E Mawji S Albendary M. Comparison of the colonic J-pouch versus straight (end-to-end) anastomosis following low anterior resection: a systematic review and meta-analysis. Int J Colorectal Dis (2022) 37:919–38. doi: 10.1007/s00384-022-04130-w 35306586

[99] Fürst A Burghofer K Hutzel L Jauch K-W. Neorectal reservoir is not the functional principle of the colonic J-pouch. Dis Colon Rectum (2002) 45:660–7. doi: 10.1007/s10350-004-6264-3 12004217

[100] Ortiz H De Miguel M Armendáriz P Rodriguez J Chocarro C. Coloanal anastomosis: are functional results better with a pouch? Dis Colon Rectum (1995) 38:375–7. doi: 10.1007/BF02054224 7720443

[101] Ho YH Tan M Seow-Choen F. Prospective randomized controlled study of clinical function and anorectal physiology after low anterior resection: comparison of straight and colonic J pouch anastomoses. Br J Surg (1996) 83:978–80. doi: 10.1002/bjs.1800830729 8813791

[102] Huber FT Herter B Siewert JR. Colonic pouch vs. side-to-end anastomosis in low anterior resection. Dis Colon Rectum (1999) 42:896–902. doi: 10.1007/BF02237098 10411436

[103] Machado M Nygren J Goldman S Ljungqvist O. Functional and physiologic assessment of the colonic reservoir or side-to-end anastomosis after low anterior resection for rectal cancer: a two-year follow-up. Dis Colon Rectum (2005) 48:29–36. doi: 10.1007/s10350-004-0772-z 15690654

[104] Laurent A Parc Y McNamara D Parc R Tiret E. Colonic J-pouch-anal anastomosis for rectal cancer: a prospective, randomized study comparing handsewn vs. stapled anastomosis. Dis Colon Rectum (2005) 48:729–34. doi: 10.1007/s10350-004-0829-z 15719189

[105] Takase Y Oya M Komatsu J. Clinical and functional comparison between stapled colonic J-pouch low rectal anastomosis and hand-sewn colonic J-pouch anal anastomosis for very low rectal cancer. Surg Today (2002) 32:315–21. doi: 10.1007/s005950200045 12027196

[106] Ramage L Mclean P Simillis C Qiu S Kontovounisios C Tan E. Functional outcomes with handsewn versus stapled anastomoses in the treatment of ultralow rectal cancer. Updates Surg (2018) 70:15–21. doi: 10.1007/s13304-017-0507-z PMC5866271 29313248

[107] Lange MM Buunen M van de Velde CJH Lange JF. Level of arterial ligation in rectal cancer surgery: low tie preferred over high tie. a review. Dis Colon Rectum (2008) 51:1139–45. doi: 10.1007/s10350-008-9328-y PMC2468314 18483828

[108] Sato K Inomata M Kakisako K Shiraishi N Adachi Y Kitano S. Surgical technique influences bowel function after low anterior resection and sigmoid colectomy. Hepatogastroenterology (2003) 50:1381–4. 14571742

[109] Irie M Kajiyama Y Enjoji A Ozeki K Ura K Kanematsu T. Changes in colonic motility in dogs after a resection of the inferior mesenteric ganglion and plexus. Surg Today (1998) 28:626–32. doi: 10.1007/s005950050195 9681612

[110] Lee WY Takahashi T Pappas T Mantyh CR Ludwig KA. Surgical autonomic denervation results in altered colonic motility: an explanation for low anterior resection syndrome? Surgery (2008) 143:778–83. doi: 10.1016/j.surg.2008.03.014 18549894

[111] Ridolfi TJ Tong W-D Takahashi T Kosinski L Ludwig KA. Sympathetic and parasympathetic regulation of rectal motility in rats. J Gastrointest Surg (2009) 13:2027–33; discussion 2033. doi: 10.1007/s11605-009-0999-z 19760300

[112] Kimura H Shimada H Ike H Yamaguchi S Ichikawa Y Kikuchi M. Colonic J-pouch decreases bowel frequency by improving the evacuation ratio. Hepatogastroenterology (2006) 53:854–7. 17153440

[113] Dinning PG Wiklendt L Gibbins I Patton V Bampton P Lubowski DZ. Low-resolution colonic manometry leads to a gross misinterpretation of the frequency and polarity of propagating sequences: Initial results from fiber-optic high-resolution manometry studies. Neurogastroenterol Motil (2013) 25:640–9. doi: 10.1111/nmo.12170 23773787

[114] Tryliskyy Y Wong CS Demykhova I Tyselskyi V Kebkalo A Poylin V. Systematic review and meta-analysis of randomized controlled trials evaluating the effect of the level of ligation of inferior mesenteric artery on functional outcomes in rectal cancer surgery. Int J Colorectal Dis (2022) 37:709–18. doi: 10.1007/s00384-022-04101-1 35152339

[115] Cirocchi R Mari G Amato B Tebala GD Popivanov G Avenia S. The dilemma of the level of the inferior mesenteric artery ligation in the treatment of diverticular disease: A systematic review of the literature. J Clin Med (2022) 11:917. doi: 10.3390/jcm11040917 35207190 PMC8880703

[116] Andreyev J. Gastrointestinal complications of pelvic radiotherapy: are they of any importance? Gut (2005) 54:1051–4. doi: 10.1136/gut.2004.062596 PMC1774900 16009675

[117] Andreyev HJN Wotherspoon A Denham JW Hauer-Jensen M. “Pelvic radiation disease”: New understanding and new solutions for a new disease in the era of cancer survivorship. Scand J Gastroenterol (2011) 46:389–97. doi: 10.3109/00365521.2010.545832 21189094

[118] Yeoh E Horowitz M Russo A Muecke T Ahmad A Robb T. A retrospective study of the effects of pelvic irradiation for carcinoma of the cervix on gastrointestinal function. Int J Radiat Oncol Biol Phys (1993) 26:229–37. doi: 10.1016/0360-3016(93)90202-7 8491681

[119] Hovdenak N Fajardo LF Hauer-Jensen M. Acute radiation proctitis: a sequential clinicopathologic study during pelvic radiotherapy. Int J Radiat Oncol Biol Phys (2000) 48:1111–7. doi: 10.1016/S0360-3016(00)00744-6 11072170

[120] Bassotti G Antonelli E Villanacci V Salemme M Coppola M Annese V. Gastrointestinal motility disorders in inflammatory bowel diseases. World J Gastroenterol (2014) 20:37–44. doi: 10.3748/wjg.v20.i1.37 PMC3886030 24415856

[121] Bassotti G Antonelli E Villanacci V Nascimbeni R Dore MP Pes GM. Abnormal gut motility in inflammatory bowel disease: an update. Tech Coloproctol (2020) 24:275–82. doi: 10.1007/s10151-020-02168-y 32062797

[122] Feeney G Sehgal R Sheehan M Hogan A Regan M Joyce M. Neoadjuvant radiotherapy for rectal cancer management. World J Gastroenterol (2019) 25:4850–69. doi: 10.3748/wjg.v25.i33.4850 PMC6737323 31543678

[123] Bondeven P Emmertsen KJ Laurberg S Pedersen BG. Neoadjuvant therapy abolishes the functional benefits of a larger rectal remnant, as measured by magnetic resonance imaging after restorative rectal cancer surgery. Eur J Surg Oncol (2015) 41:1493–9. doi: 10.1016/j.ejso.2015.07.003 26219852

[124] O’Riordain MG Molloy RG Gillen P Horgan A Kirwan WO. Rectoanal inhibitory reflex following low stapled anterior resection of the rectum. Dis Colon Rectum (1992) 35:874–8. doi: 10.1007/BF02047876 1511649

[125] Zhang B Zhao K Zhao Y-J Yin S-H Zhuo G-Z Zhao Y. Variation in rectoanal inhibitory reflex after laparoscopic intersphincteric resection for ultralow rectal cancer. Colorectal Dis (2021) 23:424–33. doi: 10.1111/codi.15444 33191594

[126] Erlandsson J Holm T Pettersson D Berglund Å Cedermark B Radu C. Optimal fractionation of preoperative radiotherapy and timing to surgery for rectal cancer (Stockholm III): a multicentre, randomised, non-blinded, phase 3, non-inferiority trial. Lancet Oncol (2017) 18:336–46. doi: 10.1016/S1470-2045(17)30086-4 28190762

[127] Keefe DMK. Gastrointestinal mucositis: a new biological model. Support Care Cancer (2004) 12:6–9. doi: 10.1007/s00520-003-0550-9 14605986

[128] Jung E Perrone EE Brahmamdan P McDonough JS Leathersich AM Dominguez JA. Inhibition of intestinal epithelial apoptosis improves survival in a murine model of radiation combined injury. PloS One (2013) 8:e77203. doi: 10.1371/journal.pone.0077203 24204769 PMC3810465

[129] Indaram AV Visvalingam V Locke M Bank S. Mucosal cytokine production in radiation-induced proctosigmoiditis compared with inflammatory bowel disease. Am J Gastroenterol (2000) 95:1221–5. doi: 10.1111/j.1572-0241.2000.02013.x 10811331

[130] Manichanh C Varela E Martinez C Antolin M Llopis M Doré J. The gut microbiota predispose to the pathophysiology of acute postradiotherapy diarrhea. Am J Gastroenterol (2008) 103:1754–61. doi: 10.1111/j.1572-0241.2008.01868.x 18564125

[131] Gerassy-Vainberg S Blatt A Danin-Poleg Y Gershovich K Sabo E Nevelsky A. Radiation induces proinflammatory dysbiosis: transmission of inflammatory susceptibility by host cytokine induction. Gut (2018) 67:97–107. doi: 10.1136/gutjnl-2017-313789 28438965

[132] Reis ED Vine AJ Heimann T. Radiation damage to the rectum and anus: pathophysiology, clinical features and surgical implications. Colorectal Dis (2002) 4:2–12. doi: 10.1046/j.1463-1318.2002.00282.x 12780647

[133] Molla M Panes J. Radiation-induced intestinal inflammation. World J Gastroenterol (2007) 13:3043–6. doi: 10.3748/wjg.v13.i22.3043 PMC4172609 17589918

[134] Beek CM Dejong CHC Troost FJ Masclee AA Troost K. Role of short-chain fatty acids in colonic inflammation, carcinogenesis and mucosal protection and healing. Nutr Rev (2017) 75:286–305. doi: 10.1093/nutrit/nuw067 28402523

[135] Corrêa-Oliveira R Fachi JL Vieira A Sato FT Vinolo MAR. Regulation of immune cell function by short-chain fatty acids. Clin Transl Immunol (2016) 5:e73. doi: 10.1038/cti.2016.17 PMC4855267 27195116

[136] Vogel I Reeves N Tanis PJ Bemelman WA Torkington J Hompes R. Impact of a defunctioning ileostomy and time to stoma closure on bowel function after low anterior resection for rectal cancer: a systematic review and meta-analysis. Tech Coloproctol (2021) 25:751–60. doi: 10.1007/s10151-021-02436-5 PMC8187190 33792822

[137] Battersby NJ Bouliotis G Emmertsen KJ Juul T Glynne-Jones R Branagan G. Development and external validation of a nomogram and online tool to predict bowel dysfunction following restorative rectal cancer resection: the POLARS score. Gut (2018) 67:688–96. doi: 10.1136/gutjnl-2016-312695 28115491

[138] Walma MS Kornmann VNN Boerma D Roos MAJ van Westreenen HL. Predictors of fecal incontinence and related quality oflife after a total mesorectal excision with primary anastomosis forpatients with rectal cancer. Ann Coloproctol (2015) 31:23–8. doi: 10.3393/ac.2015.31.1.23 PMC4349912 25745623

[139] Wells CI Vather R Chu MJJ Robertson JP Bissett IP. Anterior resection syndrome–a risk factor analysis. J Gastrointest Surg (2015) 19:350–9. doi: 10.1007/s11605-014-2679-x 25326125

[140] Liu F Guo P Shen Z Gao Z Wang S Ye Y. [Risk factor analysis of low anterior resection syndrome after anal sphincter preserving surgery for rectal carcinoma]. Zhonghua Wei Chang Wai Ke Za Zhi (2017) 20:289–94. 28338162

[141] Ihn MH Kang SB Kim DW Oh HK Lee SY Hong SM. Risk factorsfor bowel dysfunction after sphincter-preserving rectal cancer surgery: aprospective study using the memorial Sloan Kettering cancer centerbowel function instrument. Dis Colon Rectum (2014) 57:958–66. doi: 10.1097/DCR.0000000000000163 25003290

[142] Beamish EL Johnson J Shaw EJ Scott NA Bhowmick A Rigby RJ. Loop ileostomy-mediated fecal stream diversion is associated with microbial dysbiosis. Gut Microbes (2017) 8:467–78. doi: 10.1080/19490976.2017.1339003 PMC5628638 28622070

[143] Erickson JC Bruce LE Taylor A Richman J Higgins C Wells CI. Electrocolonography: Non-invasive detection of colonic cyclic motor activity from multielectrode body surface recordings. IEEE Trans Biomed Eng (2019); 67(6):1628–37. doi: 10.1109/TBME.2019.2941851 31535980

